# Effect of infrared belt and hot water bag on labor pain intensity among primiparous: a randomized controlled trial

**DOI:** 10.1186/s12884-023-05689-0

**Published:** 2023-06-01

**Authors:** Fatemeh Dastjerd, Fatemeh Erfanian Arghavanian, Ameneh Sazegarnia, Farideh Akhlaghi, Habibollah Esmaily, Masoumeh Kordi

**Affiliations:** 1 School of Nursing and Midwifery, Mashhad University of Medical Sciences, Mashhad, Iran; 2 Nursing and Midwifery Care Research Center, Mashhad University of Medical Sciences, Mashhad, Iran; 3 Department of Midwifery, School of Nursing and Midwifery, Mashhad University of Medical Sciences, Mashhad, Iran; 4 Department of Medical Physics, Medical Physics Research Center, Faculty of Medicine, Mashhad University of Medical Sciences, Mashhad, Iran; 5 Department of Obstetrics and Gynecology, Faculty of Medicine, Mashhad University of Medical Sciences, Mashhad, Iran; 6 Department of Biostatistics, School of Health, Mashhad University of Medical Sciences, Mashhad, Iran

**Keywords:** Thermotherapy, Labor pain, Infrared rays, Complementary therapies

## Abstract

**Background:**

Labor pain is complex, paradoxical and varied in every parturient woman. Management of labor pain has been a crucial component in maternity care. Heat therapy is one of the proposed method for labor pain relief. Infrared is one of the methods of heat therapy but there is any study in this regard. This study aimed to compare the effect of the infrared belt and hot water bag on the severity of pain in the first stage of labor among primiparous women.

**Methods:**

In this clinical trial in the first stage of labor, 20-min cycles of heat therapy were conducted at the dilations of 4–5 and 6–7 cm in the intervention group 1 by an infrared belt and in the intervention group 2 by hot water bag, respectively. The control group received routine care. The severity of the pain was measured by the short-form McGill Pain Questionnaire.

**Results:**

In total, 136 women consented to participate in this clinical trial study. The mean score of pain intensity was significantly lower in the two intervention groups compared to the control group (*P* < 0.001). The mean pain intensity was significantly lower in the infrared belt group than in the hot water bag group (*P* < 0.001).

**Conclusions:**

Based on these findings, heat therapy with an infrared belt reduced the severity of pain in the first stage of labor. The infrared belt could be used and recommended as a safe and effective pain relief in childbirth and maternity care.

**Trial registration:**

This study was registered in the Iran Clinical Trial Center with the code of IRCT20190805044446N1.

## Background

The labor and its related pain are a unique experience for every parturient woman. The majority of women experience pain during childbirth as a normal physiological aspect of this process. Labor pain is complex and paradoxical which is different and diverse in women. Generally, every woman experiences it in different ways from excruciating to a pleasure [1–3]. The severity of labor pain is influenced by physical, psychological, mood, cultural, and ethnic factors. Regarding this, it has been found that age, obesity, number of deliveries, maternal and fetal status, fetal to pelvic size ratio, maternal status during labor, history of a difficult child birth, maternal fatigue and psychological factors like fear and anxiety aggravate maternal pain [4–6]. These factors may be contributed to the increase use of intervention [1], so the management of labor pain is a challenging and important part of care during the childbirth [2, 6].

In this regard pharmacological and non-pharmacological methods can be applied to relieve labor pain. Pharmacological methods for pain relief include; Inhaled analgesia, Opioid and Non-opioid drugs, Local anesthesia and Epidural [7–9]. However, these types of methods are associated with side effects for both mothers and neonates [9, 10]. For this reason, non-pharmacological methods have been taken into consideration. As they are non-invasive, seems to be safe for the mother and fetus and bring a pleasure experience for parturient women [8, 11, 12].

Among this method, physical therapy involves the use of heat, cold, hydrotherapy, manipulation, and electric currents. Heat therapy is one of the most common methods used in physical therapy [13]. Heat can be used superficially in the form of infrared (IR), hot water bag, electric heating pad, and silicone gel heating pad; or it can be applied deeply in the form of ultrasound or diathermy [5, 6, 14].

Heat causes the blood vessels to dilate and increases the blood flow, which will lead to a growth in the volume of cell exchange. Stimulation of heat receptors, based on the pain gate mechanism, closes the pain transfer valves, controls the entry of pain impulses into the nerve pathways, consequently, reduces the pain [5, 14].

Silicone hot water bags, which heat the under-treatment area and increase the blood circulation in that region are used to reduce spasm, pain, and soft tissue stiffness [14]. Numerous studies have examined the effect of heat therapy on pain intensity at different stages of labor, showing the positive effect of this type of therapy on pain intensity in labor and child birth [15–20].

Another method of heat therapy is using IR [14]. Infrared thermal radiation is categorized as a subset of electromagnetic, non-ionizing, short-wavelength waves that have been used effectively for pain reduction and treatment in some disease over the years [21, 22].

It was reported that infrared radiation absorption activates signaling pathways involved in pain perception. Potential explanations for how infrared therapy might relieve musculoskeletal pain include increased nitric oxide (NO) levels, decreased oxidative stress, and the induction of inflammatory mediators [23].

Through local processes, oxidative stress may change nociception and result in hyperalgesia. Reactive oxygen species produced as a result of oxidative stress damage tissues and induce inflammation, which in turn stimulates sensory neurons that are involved in the transmission of pain more intensely. Infrared therapy has the potential to reduce pain and inflammation in the muscles by lowering the degree of oxidative stress [24].

Numerous investigations have shown that the biological effects of infrared irradiation are connected to the NO pathway. Vasodilation, which decreases laminar shear stress, is one of the short-term effects of infrared irradiation. It has been demonstrated that NO has a role in peripheral inflammatory and neuropathic pain. The activation of ATP-sensitive K + channels in the membrane of nociceptors, is one proposed mechanism of action of NO as a peripheral antinociceptive agent [25, 26].

Some studies revealed that electrically and noxiously elicited action potentials, including pro-inflammatory mediators, were inhibited by infrared light. Microtubule array disruption and rapid axonal flow may support neuronal inhibition [27].

To the best of our knowledge, no specific research has been conducted to investigate the effect of infrared belt on labor pain. However, some studies have been performed evaluating the effect of IR on pain relief of primary dysmenorrhea. In this regard, Yi Liau et al. (2011) and Lee et al. (2009) showed that infrared belt can be used as an appropriate and effective way for the treatment the symptoms and relief of dysmenorrhea [28, 29]. Moreover, Ansari et al. (2013) and Gale et al. (2006) reported that IR therapy have positive effects on reducing pain in patients with low back pain [30, 31]. In line with mentioned studies, in a study by Ammar TA (2015), it was reported that monochromatic infrared photo energy may play a role in treating chronic low back pain [32]. Moreover, in a study by Siems W. et al. in the group of patients with low back pain affected by infrared light, rapid beneficial effects of infrared light towards musculoskeletal pain and joint mobility loss were demonstrated [33]. Another study reported that pain sensitivity of myofascial trigger points in chronic non-specific low back pain is improved by linear polarized polychromatic light treatment in the red and near-infrared spectrum [34].

Exposure to infrared radiation is not dangerous and poses no significant risk, because, infrared photons are not powerful enough to produce ionizing radiation [35]. The infrared light source is considered safe for the fetus as it has not any direct contact with the fetus's eyes, is non-ionized, and disappears before reaching the fetal layers. According to the International Commission on Non-Ionizing Radiation Protection, the permissible light intensity from the mother's abdomen is set at 48.3 mw (502 mw/cm2) and 86.9 mw (904 mw/cm2) for continuous and intermittent radiation, respectively [35, 36].

To the best of our knowledge, no particular research has been carried out comparing infrared belt with hot water bags in labor pain relief. Therefore, this study was designed and conducted to evaluate the effect of the infrared belt and the hot water bag on the severity of labor pain in primiparous women and report the possible side effects of these methods.

## Methods

### Trial design

The study design was a randomized controlled trial and recruited three groups of primiparous women birthing at Umm Al-Banin Hospital, Mashhad, Iran, from July 2019 until October 2019.

### Participants

The women were selected from the patients referring to the maternity ward of the hospital.

The inclusion criteria included; 1) women's age range of 18–35 years, 2) primiparity, 3) single birth, 4) cephalic presentation with no Cephalopelvic Disproportion (CPD), 5) maternal age within the range of 38–42 weeks, 6) estimated fetal weight between 2500–4000 g according to Johnson's law or ultrasound after 32 weeks, 7) Spontaneous onset of labor 8) labor pain intensity of ≥ 3 based on the visual analog scale for pain (10 cm ruler), 9) no drug addiction during the past year, 10) no medical illnesses, 11) no obstetric complications, 12) no history of surgery on the uterus or cervix, 13) maternal oral temperature of < 38 °C, 14) no severe anxiety, 15) no lesion in the lumbar region.

On the other hand, women with the following criteria were excluded from the study; emergency cesarean section, the use of oxytocin to induce or accelerate labor, the use of pain relief medication like narcotics, Entonox, or anesthesia, symptoms regarding fetal distress, disturbance in labor process, the observation of any possible complications related to infrared belt and those who were unwilling to continue their collaboration.

In this study, out of 150 participants, 14 women were excluded from the study, including 5 patients in the infrared belt group(2 for receiving pethidine and Entonox, 2 for cesarean section due to fetal distress and lack of progress respectively, 1 for unwillingness to continue the intervention), 5 patients in the hot water bag group (2 for lack of progress and cesarean section, 1 for stopping the intervention, and 2 for receiving pethidine), and 4 women in the control group(3 for cesarean section due to fetal distress and lack of progress, 1 for receiving pethidine and Entonox).

Eventually, the analysis was performed on the data obtained from 136 cases, including 45, 45, and 46 cases in the intervention 1, intervention 2, and control groups, respectively. The results of the study were compared before and after the intervention in the three groups.

### Interventions

Infrared belt: Infrared belt used in this study was made of tourmaline ceramic stones (79 pieces) with dimensions of 135 × 18 cm and a weight of 1.85 kg (Fir Bio Photon Led Light Electric tourmaline heating waist belt, Liaoyang Conoval Technology Co, China). This belt is capable of working 8 h/day with a temperature range of 37–70 °C, which was used with a temperature range of 38–40 °C, wave length of 850 nm and an intensity of 15 mW/cm2 in this study. The validity and reliability of this belt were confirmed in Bu Ali Research Institute, Mashhad, Iran, by a laser thermometer (Extech, USA) at room temperature of 25.5 °C and with an IR camera (TESTO, Germany).

Hot water bag: A silicone bag with a cloth cover filled with warm water. First, the water bag was filled with desired-temperature water. Afterward, the water temperature was set at 38–40 °C by placing a mercury thermometer between the bag and the cloth cover. The reliability of the hot water bag was determined at each use by a mercury thermometer placed between the hot water bag and the cloth cover.

In intervention group 1, pain intensity in cervical dilatation of 4–5 cm was measured with the short-form of McGill Pain Questionnaire. Before the intervention, the temperature of infrared belt was adjusted, and then, was fastened around the women's waist lying on the left side for 20 min. During the intervention, the fetal heartbeat was being monitored. After 20 min the belt was unfastened and the pain intensity was measured again. The researcher performed a vaginal examination every hour. The second cycle of intervention started at dilation of 6–7 cm and was performed for 20 min. The pain intensity was measured again at the end of this phase. Afterward, the researcher measured the labor pain severity at the end of the first stage of labor (10 cm dilation of the cervix).

In intervention group 2, pain intensity was measured in cervical dilatation of 4–5 cm using the short-form McGill Pain Questionnaire. The intervention was performed in two 20-min cycles with a hot water bag. First, the hot water bag, covered with a cloth cover, was filled with hot water and its temperature was set at the range of 38–40 °C by placing a mercury thermometer between the hot water bag and the cloth cover.

Subsequently, while the mother was lying on her left side, the researcher placed the hot water bag on her waist (dilatation of 4–5 cm) for 20 min. At the end of 20 min, pain intensity was reassessed using the McGill questionnaire. The fetal heart rate was monitored during the intervention. The second cycle of the intervention was performed at a dilation of 6–7 cm for 20 min, at the end of which the pain intensity was evaluated again. Afterward, the researcher measured the labor pain severity at the end of the first stage of labor (10 cm dilation of the cervix).

In the control group, the researcher was present at the delivery bed from cervical dilatation of 4–5 cm and performed routine care. The pain intensity was measured using the McGill questionnaire in the cervical dilatations of 4–5 cm and 6–7 cm and at the end of the first stage of labor.

The researcher was present at the delivery bed of patients in the three groups until the end of the second stage.

### Outcome measures

The instruments used in this study included a research unit selection checklist, demographic-pregnancy form, and the short-form McGill Pain Questionnaire. Moreover, three information forms were also employed to gather data, one of which was related to the progress of labor and fetal and maternal status, the other one was related to the first and second stages of labor and infant status and the last one regarding women's satisfaction from their intervention. Also fatigue and hunger was measured through two separate visual analog scale for pain(100-mm line at the end of which means not having fatigue or hungry and 100 at the other end indicates the most severity of fatigue or hungry respectively). Spielberger state- trait anxiety inventory has been used as anxiety measurement as well. The validity of the questionnaires (demographic, progress of labor, fetal and maternal status, first and second stages of labor and satisfaction from intervention) was determined using content validity by the first researcher of paper. The application of the short-form McGill Pain Questionnaire, as a valid tool for pain measurement, has been approved in different countries. It has also been validated in Iran by using criterion validity with an obtained correlation of 0.854. The reliability of this instrument has been confirmed repeatedly in different studies performed in Iran and other countries [37, 38]. In this study, the reliability of this tool was estimated at 0.96 using the consistency of evaluator’s method. The fatigue and anxiety and STAI were the standard questionnaire, that in our study their reliability were assessed through consistency of evaluators method (*r* = 0.96, *r* = 0.98 respectively) and internal consistency method with Cronbach's alpha coefficient 0.99 for the STAIS.

### Sample size

The women were selected from the patients referring to the maternity ward of the hospital using the convenience sampling method. Afterward, women were assigned into three groups, namely infrared belt, hot water bag, and control (routine care), using the block randomization method. To determine the sample size, the results of the pilot study were applied on ten women in three group, and the formula of comparing two means was used to estimate the pain score. The sample size of at least 45 women in each group was calculated, in which the final sample size was determined at 50 women in three groups based on 80% test power, 95% confidence interval, 5% maximum margin of error, and 10% drop-out rate. It should be mention that the results of the pilot study were not used in the final analysis.

### Blinding

No blinding was performed in this study, and to prevent any bias in the results, the same duration of heat therapy was considered in both study groups.

### Statistical analysis

The forms were coded and entered into the computer, followed by ensuring the accuracy of data entry through its control by the second person. The collected data were analyzed in SPSS software (version 25). The Kolmogorov–Smirnov test was first used to investigate the normal distribution of quantitative variables. To this end, the homogeneity of the three groups was examined in terms of quantitative and qualitative variables by Chi-square, Kruskal–Wallis, one-way analysis of variance and Fisher's exact tests. Since a 95% confidence interval was considered in all tests, the *p*-values of < 0.05 were reported to be significant.

### Ethical considerations

This study was approved by the Ethics Committee of Mashhad University of Medical Sciences (IR.MUMS.NURSE.REC.1398.029), Mashhad, Iran. Written, signed, and informed consent was obtained from all participants in the study. It has been confirmed that all methods were carried out in accordance with relevant guidelines and regulations. The participant’s information was kept confidential and this clinical trial was conducted in adoptive design and the patients were informed that they are permitted to leave the trial whenever they want without any reason. They also were monitored during the study to check about the probable adverse effects of the intervention e.g. burning, allergic rash and skin reaction at the location of the belt and hot water bag and in case of occurrence were managed or removed from the case group.

This clinical trial was registered in national registry for clinical trials and approved with this ID IRCT20190805044446N1.

## Results

**Fig. 1:**
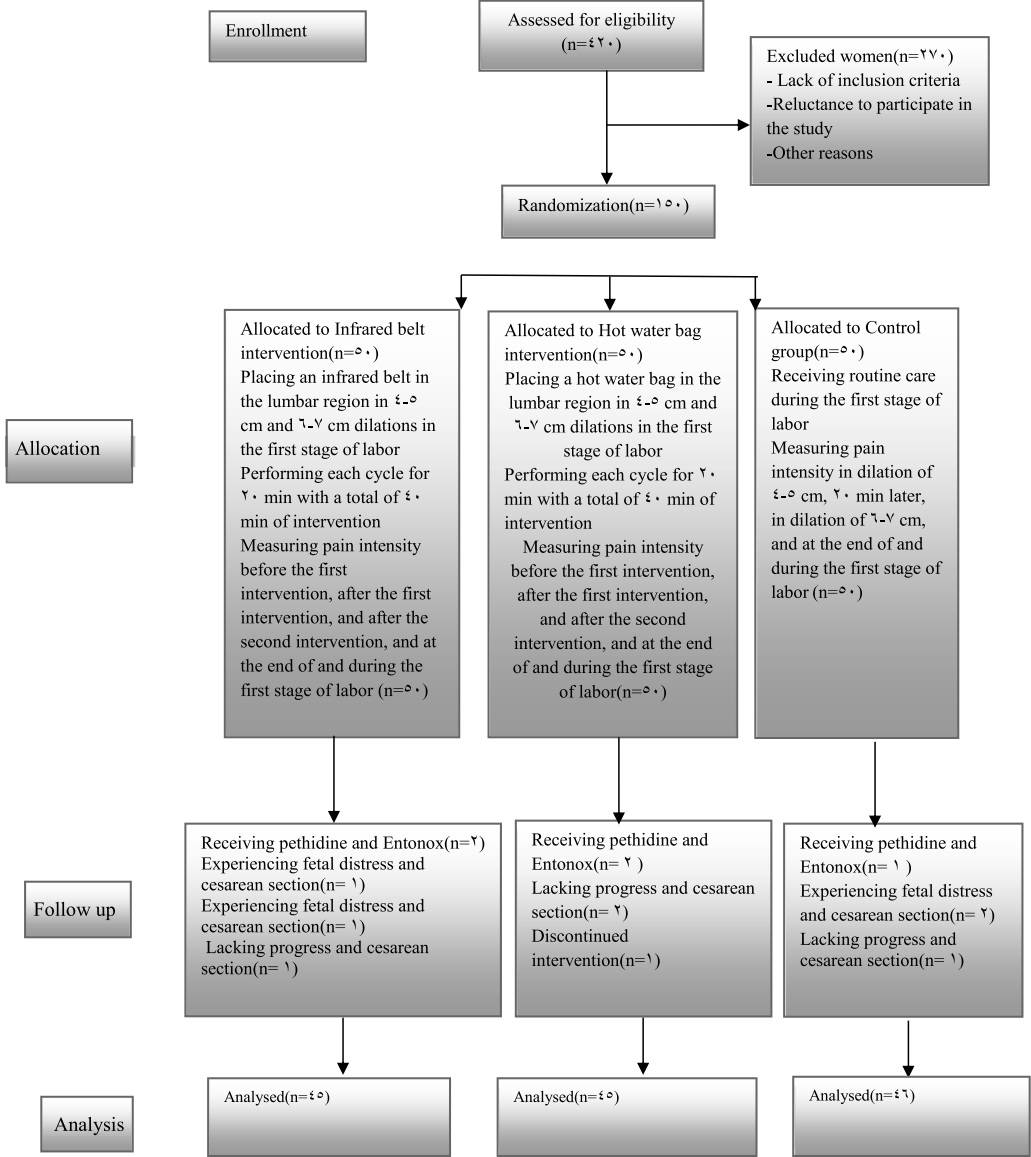
The CONSORT flow diagram of intervention in the three groups

In this clinical trial study 136 women consented to participate (see Fig. 1 flow chart). The mean ages of women in the three groups; infrared belt, hot water bag and control did not differ significantly (*P* = 0.42). Moreover, no significant difference was observed in the maternal and paternal education levels in the three groups (*P* = 0.05 and *P* = 0.54, respectively) (Table 1).

**Table 1:** Mean score of maternal age, gestational age, fatigue and hunger in the three groups at the baseline

Variable	Infrared belt group	Hot water bag group	Control group	Test results
	Mean ± SD	Mean ± SD	Mean ± SD	
Maternal age(year)	24.78 ± 4.53	23.76 ± 4.65	23.98 ± 4.71	Χ^2^ = ^*^1.72 *P*-value = 0.42
Gestational age	39.83 ± 0.79	39.63 ± 0.73	39.38 ± 0.93	*Χ^2^ = 7.05 *P*-value = 0.37
Fatigue score at admission	57.19 ± 23.7	50.83 ± 25.81	58.71 ± 24.96	*Χ^2^ = 2.83 *P*-value = 0.24
fear	56.32 ± 10.06	54.3 ± 14	53.28 ± 13	*Χ^2^ = 2.97 *P*-value = 0.22
number of uterine contractions	2.4 ± 0.4	2.34 ± 0.41	2.44 ± 0.44	F = 10.48 *P*-value = 0.79
Hunger score at admission	38.81 ± 24.35	31.95 ± 22.66	25.12 ± 22.39	*Χ^2^ = 6.11 *P*-value = 0.04
anxiety	47.23 ± 8.05	41.98 ± 9.08	42.2 ± 8.51	F = 9.11 *P*-value < 0.001

^*^Kruskal–Wallis

Based on the results, the mean gestational age was homogeneous in the three groups and was not significant (*P* = 0.37). Pregnancy care was sufficient for most participants and no significant difference was revealed in the three groups (*P* = 0.54).

According to Table 1, the mean score of fatigue, fear, and the number of uterine contractions was not significantly different in the three groups and the groups were homogeneous regarding these variables (*P* = 0.24, *P* = 0.22, and *P* = 0.79, respectively).

However, the mean score of hunger and anxiety showed a significant difference in the three groups (*P* = 0.04 and *P* < 0.001, respectively). The scores of these variables at the baseline were considered as confounding variables.

The results of the McGill Pain Questionnaire (regarding mean scores of pain intensity) were not significantly different in the three groups at the baseline (*P* < 0.13). But there was a difference after the first and second interventions and at the end of the first stage of labor in the three groups, compared to those at the baseline (*P* = 0.001, *P* < 0.001, and *P* < 0.001, respectively).

It was reported that the mean total score of pain, based on the McGill questionnaire, was significant in the first stage of labor in the three groups, compared to that before the intervention (*P* < 0.001) (Table 2).

**Table 2:** Mean scores of McGill pain score in the three groups

Variable	Infrared belt group	Hot water bag group	Control group	Test results
	Mean	Mean	Mean ± SD	
Score of Pain at admission	43.73 ± 6.9	42.93 ± 6.98	23.98 ± 4.71	**f = 202.31 *P*-value = 0.13
Score of Pain after first intervention	25.91 ± 5.48	35.73 ± 7.53	39.38 ± 0.93	*Χ^2^ = 82.52 *P*-value < 0.001
Score of Pain after second intervention	27.04 ± 6.94	39.07 ± 6.52	58.71 ± 24.96	*Χ^2^ = 82.28 *P*-value < 0.001
Score of Pain at the end of first stage of labor	38.49 ± 7.28	45.6 ± 6.71	53.28 ± 13	*Χ^2^ = 54.17 *P*-value < 0.001
Mean score of total pain in the first stage of labor(after study enrollment)	30.48 ± 6.5	40.1 ± 8.41	2.44 ± 0.44	*Χ^2^ = 67.21 *P*-value < 0.001

^*^Kruskal–Wallis

^**^One-way analysis of variance

The two-by-two comparison of the groups using Tukey's and Dunn(Bonferroni correction) multiple comparison tests showed that the pain intensity score in the first stage of labor was significantly different immediately after both the first and second interventions in the infrared belt and hot water bag groups, compared to that in the control group (*P* < 0.001).

Furthermore, pain intensity was significantly lower in the infrared belt group immediately after the first and second interventions than in the hot water bag and control groups (*P* < 0.001).

Based on the results obtained from the two-by-two comparison of the groups using Tukey's and Dunn(Bonferroni correction) multiple comparison tests, the pain intensity score in the first stage of labor was significantly different in the infrared group than in the control group (*P* < 0.001).

It was also reported that the intensity of pain at the end of the first stage of labor was significantly lower in the infrared belt and hot water bag groups than in the control group (*P* < 0.001, *P* = 0.002, respectively).

The two-by-two comparison of the groups using Tukey's and Dunn(Bonferroni correction) multiple comparison tests showed that the mean total scores of McGill pain in the first stage of labor was significant in the infrared belt and hot water bag groups, compared to the control group, before the intervention (*P* < 0.001).

Additionally, the McGill total pain score in the first stage of labor was significantly lower in the infrared belt group than in the hot water bag and control groups (*P* < 0.001).

As reported in Table 3, the statistical tests of analysis of covariance (NCOVA) revealed significant differences between the groups in terms of the pain intensity. So that, with the control of the Pain at admission, anxiety and hunger the pain after the first intervention in the infrared belt and hot water bag was significantly lower compare to the control group (it was decreased 19 and 7.43 respectively). After control the confounder variables, the pain was significantly lower in in the second (it was decreased 21.63and 8.19respectively) and at the end of the first stage of labor (it was decreased 13.11and 5 respectively) in the infrared belt and hot water bag compare to the control group (Table 3).

**Table 3:** Result of analysis of covariance on the effect of intervention on pain intensity with control the confounder variables

Dependent Variable	Parameter	B	Std. Error	t	Sig
Score of Pain after first intervention	Infrared belt group	-19.000	.932	-20.377	< 0.001
	Hot water bag group	-7.434	.884	-8.407	< 0.001
	Control group	0^a^			
	Score of Pain at admission	.649	.054	12.087	< 0.001
Score of Pain after second intervention	Infrared belt group	-21.632	1.199	-18.037	< 0.001
	Hot water bag group	-8.192	1.137	-7.202	< 0.001
	Control group	0^a^			
	Score of Pain at admission	.488	.069	7.075	< 0.001
Score of Pain at the end of first stage of labor	Infrared belt group	-13.114	1.385	-9.468	< 0.001
	Hot water bag group	-5.009	1.313	-3.814	< 0.001
	Control group	0^a^			
	Score of Pain at admission	.310	.080	3.894	< 0.001

^a^refrence

The three groups had significant difference in terms of progression of labor in first (*P* = 0.002) and second (*P* = 0.002) stage of labor and infrared belt group had higher normal labor progress.

The majority of women participating in this study reported their satisfaction degree with the hot water bag and infrared belt as good (56.8%) and very good (86.0%), respectively and women participated in infrared belt group had more satisfaction. It is worthwhile to report that 76% and 51% of women recommended their intervention (infrared belt and hot water bag respectively) to others. Their reason in the infrared belt group was as: it has a fine and pleasant warmth (18%), It reduces the pain very much (8%) and reduces the pain (22%). In the hot water bag group, women`s reason was as: It makes good sense (22%) and it reduces the pain a little (27%).

Also, the three group hadn`t significant difference in terms of 1 (*P* = 0.47) and 5 (*P* = 0.6)-min Apgar scores. There was no adverse effect like abnormal fetal heart rate and PPH in the two-intervention group. No skin reaction and hyperthermia or any other adverse effect were reported in the infrared belt and hot water bag group.

## Discussion

The results of the present study showed that the pain intensity at the first stage of labor was lower, in the hot water bag and infrared belt groups than in the control group, while it was lower in the infrared belt group compared to the hot water group.

Behmanesh et al. (2009) reported that the pain intensity of the first stage of labor was lower in the hot water bag group than in the routine care group [15]. Based on the results of a study carried out by Ganji et al. (2011), the severity of labor pain was lower in the alternating hot and cold therapy group than in the control group in different stages of labor [16]. Other studies have been reported that the heat therapy has been effective in reducing the intensity of labor pain in different stage as well [17–19] which confirm the results have been obtained from the present study.

The mechanism of heat in alleviating pain is well explained in the Gate Control Theory of Pain [16]. Regarding, when heat enters into a body region, the touch and temperature receptors are stimulated and compete with the pain receptors. Temperature and touch signals reach the spinal cord faster than the pain signals, in which alleviate the pain [19]. Another mechanism for the pain relief through the heat application is releasing endorphins [39].

To the best of our knowledge, no specific study has been performed to investigate the effect of an infrared ceramic belt on labor pain. However, the results of other studies were indicative of the effectiveness of infrared in alleviation of pain like dysmenorrhea [28, 29] and low back pain [30, 31]. Also in line with our study, another research was conducted in order to assess the effect of light-emitting diode (LED) photobiomodulation on analgesia during labor by LED plate with red and infrared merged [red 660 ± 20 nm, 5 mW/cm2, 3 J per LED (108 J) and infrared 850 ± 20 nm, 5 mW/cm2, 3 J per LED (108 J), total energy = 216 J] and it was reported that LED can be viewed as an alternative to hot showers since it reduced discomfort during delivery without altering other factors, making it a strategy that is safe enough to be employed in hospital protocols [40].

This study was the first clinical trial regarding using the ceramic belts with infrared merged, as a non-pharmacological method and complementary medicine, which was safe and effective in relieving labor pain.

One of the possible mechanisms of infrared radiant ceramic belts is related to the infrared thermal effects which radiated from ceramic stones (natural minerals are infrared radiant) [41]. Infrared can penetrate into the deep tissues through the skin and transfer energy to deep tissues through the mechanism of absorption by organs and water molecules without irritating the skin excessively [42].

The other possible mechanism is that infrared is effective in inhibiting prostaglandin production and has anti-inflammatory and analgesic effects. Also, infrared ceramic materials exert an antioxidant effect by increasing the degradability of hydrogen peroxide, which consequently, reduces ischemia and pain [43]. This matter could be another reason for usefulness of infrared in relieving labor pain. Since hypoxia myometrium is one of the main causes of labor pain and prostaglandin has a role in this process [4].

Another point is that, infrared radiation increases calcium-dependent nitric oxide (NO) [44, 45]. NO is an essential substance for feminine cells, such as uterine cells. NO activity can improve peripheral blood circulation and is effective in myometrial relaxation and relieving myometrial contractions resulted effective contraction and progressive labor [46].

Also the biological and physiological effects of infrared are not only related to heat but also to the main types of light receptors(i.e., cytochrome oxidase and intracellular water), in other word infrared has thermal and radiant effect as well [21, 22].

Yaghoubi et al. (2012) conducted a study to compare the effect of infrared and hot water bag on lumbar disc herniation pain represented that the pain intensity was decreased equally in both groups [14]. In the present study, the temperature of heat therapy was 38–40 °C but in the above mentioned study it was 70° C, and the infrared was implemented by using a 250-W lamp. The discrepancy in results of these two studies can be attributed to the differences in the temperature adopted in the heat and infrared therapies. Another matter is that the extent of temperature transfer is directly related to temperature, exposed area, and duration of use [15]. Hot pack raises the skin temperature by at least one centigrade degree and penetrates to a depth of 1 cm below the skin [47]. However, heat penetration depth should be 3–5 cm to increase tissue flexibility [48]. Temperature penetration depth in infrared thermotherapy is reported to be 3–4 cm [21, 49].

The results of the present study showed no difference in the 1- and 5-min Apgar scores between the intervention and control groups. In Akbarzadeh et al. (2017) study, there was no difference between the hot compress group and the routine group regarding the 1- and 5-min Apgar scores as well [50], which was in line with the results of the current study.

The results of the present study showed that there was a significant difference between the two intervention groups in terms of satisfaction and infrared belt had greater effect in this regard. In Taavoni et al. (2013) study the mean score of satisfaction was significantly higher in the heat therapy group than in the control group as well [17] which was in line with the results of the present study.

It has been reported when women's expectations of childbirth are met, their satisfaction would be increases. Some of the aspect of labor like relieving pain and reducing labor length is related to women`s satisfaction. The greater satisfaction after intervention with the infrared belt can be explained by the greater effectiveness, reduced labor length and ease of use [17, 51].

As a strength of our study, it is worthwhile to mention that this study is the first clinical trial which investigate the effect of infrared belt as a new non pharmacologic method on the management of labor pain. This random allocation study was performed on three groups which compare the effect of infrared belt with hot water bag on the labor pain intensity. Women’s difference in the pain tolerance and other characteristic like their cognitive attributes can be affected on their pain response, which was controlled by random allocation. Moreover, environmental conditions, could be influenced the pain intensity of women, which was the same in all groups (similar sampling environment).

## Limitations of the study

The difference in the pain tolerance threshold of the research units has an effect on the response of people to the pain relief method, which was controlled by random allocation. Environmental conditions such as the behavior of the staff in the department and physical conditions such as sound, light and temperature had an effect on the pain intensity of the research units, and for this reason, the sampling environment was chosen the same in all groups.

## Conclusion

The results of the present study showed that thermotherapy with an infrared ceramic belt and hot water bag was effective in reducing pain in the first stage of labor. It was also revealed that the pain intensity of the first stage of labor was lower in the infrared belt group than in the hot water bag. Therefore, infrared belt, as a complementary method is more effective than the hot water bag in reduction the labor pain intensity. Based on the results of the present study it is recommended the use of infrared belts over the hot water bags as a safe and effective pain relief in maternity care, the birthing center and maternity wards, to improve the quality of women's care and bring a more satisfactory experience for women during childbirth.

## Data Availability

The datasets used and/or analysed during the current study are available from the corresponding author on reasonable request.
